# Oxidative stress in elderly population: A prevention screening study

**DOI:** 10.1002/agm2.12121

**Published:** 2020-08-09

**Authors:** Davide Gorni, Annarosa Finco

**Affiliations:** ^1^ Department of Oxidation Research Cor. Con. International Srl Parma Italy

**Keywords:** aging, antioxidant, d‐ROMs test, oxidative stress, peroxides

## Abstract

**Background:**

Aging is a multifactorial phenomenon, characterized by a progressive decline in the efficiency of biochemical and physiological processes and an increased susceptibility to disease. There is increasing evidence that aging and age‐related disease are correlated with an oxidative stress (OS) condition. The latter is characterized by an imbalance between reactive species (RS), in particular reactive oxygen species (ROS) and antioxidant reserve.

**Objectives:**

The aim of this study is to evaluate the two main markers of oxidative stress, plasmatic peroxide concentration (through d‐ROMs FAST test, derivates‐Reactive Oxygen Metabolites) and plasmatic antioxidant power measured by iron‐reducing power (PAT test, Plasma Antioxidant Test) in 290 apparently healthy volunteers over 60, and their possible correlation with age and gender.

**Materials and methods:**

Human capillary blood samples from healthy volunteers were used in this observational study for the evaluation of the markers of OS.

**Results:**

The data obtained broadly demonstrate that the majority of elderly people display an OS condition characterized by increased levels of peroxides and a slight reduction in antioxidant reserve.

**Conclusions:**

Seniors have a greater propensity to develop a condition of oxidative stress, and therefore it is important to associate the monitoring of oxidative stress markers and, if necessary, antioxidant supplementation, with a healthy lifestyle.

## Graphical abstract

Different factors involved in healthy aging.

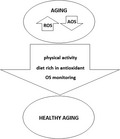

## 1 INTRODUCTION

Aging is characterized by a progressive decline in the efficiency of biochemical and physiological processes, the functional maintenance of tissue homeostasis and an increasing susceptibility to diseases.

Aging is a multifactorial process, which is genetically determined and influenced epigenetically by environment.^1^

In recent years it has been well known that oxidative stress may play important roles in elderly health and, in particular, there is increasing evidence that aging might be caused by the potential and harmful effects of an accumulation of oxidative damage caused by reactive species (RS, in particular reactive oxygen species or ROS and reactive nitrogen species RNS). This evidence is supported by the “free radical theory of aging” proposed by Harman.^2^

Normal levels of RS are essential for various cellular mechanisms.^3^, ^4^, ^5^ High levels of ROS/RNS would be detrimental to cells and have been thought to contribute to aging and the pathogenesis of numerous age‐related diseases.^4^, ^6^ Antioxidant reserve (RAO) controls the production of ROS.^7^

*Reactive oxygen species*, in particular hydrogen peroxide (H_2_O_2_) and superoxide anion ($O_{2}^{\cdot ‐}$), are physiologically produced in a number of cellular reactions, including the iron‐catalyzed Fenton reaction,^8^, ^9^ and by various enzymes, such as lipoxygenases, peroxidases, NADPH oxidase and xanthine oxidase.

An excess of oxidation and/or a lack of antioxidant cause a condition of oxidative stress (OS).^7^ The OS condition can be temporary or prolonged depending on the response of the antioxidant defense network. A prolonged OS condition can cause damage to all the macromolecular constituents of the body such as lipids, protein and DNA^10^, ^11^ and is ultimately an emerging risk factor for human health and for the development of many diseases (diabetes, cancer, cardiovascular and neurodegenerative diseases, aging).^7^, ^12^, ^13^, ^14^, ^15^, ^16^, ^17^

*Reactive oxygen species* are generated mainly as byproducts of mitochondrial respiration; mitochondria are the primary target of oxidative damage and play an important role in aging. Emerging evidence has linked mitochondrial dysfunction to a variety of age‐related diseases, including neurodegenerative diseases and cancer.^18^

For example, in the aging brain and in the case of several neurodegenerative diseases, there is a decline in the normal antioxidant defense mechanisms, which increases the vulnerability of the brain to the deleterious effects of oxidative damage.^19^

The main aim of this observational study is the evaluation of the two main markers of oxidative stress in a population over 60 and their possible correlation with age and gender. These markers allow the plasma blood concentration of peroxides to be determined, as the oxidative damage index, and the plasma blood iron‐reducing capacity, as antioxidant reserve index.

## 2 METHODS

### 2.1 Recruitment and type of study

Awareness‐raising sessions for senior citizens (over 60) were organized by National Associations of Categories on the topic of oxidative stress and aging. Volunteers were asked to provide a plasma sample free of charge for the evaluation of their global oxidative stress. All subjects were informed and asked to sign an informed consent form to authorize the use of the data obtained from the analysis of oxidative stress markers, in anonymous form, for research purposes. They were also asked to provide a declaration of absence/presence of chronic disease and pharmacological therapies. Appropriate anonymization methods were applied. The analyses were carried out by the same operator.

### 2.2 Volunteer details

Two hundred and ninety volunteers took part in the study: 105 men and 185 women between the ages of 60 and 92 were analyzed after giving written and informed consent.

The exclusion criteria were as follows: age under 60, recent trauma, recent or ongoing viral or bacterial infection. Capillary plasma was obtained by puncture the fingertip of the middle finger in resting conditions. None of them was taking antioxidant supplements.

### 2.3 Type of study

The oxidative balance was measured through a single determination using dedicated tests. The concentration of peroxide, marker of lipid oxidation and the ferric reducing capacity as total antioxidant capacity were measured in blood plasma samples.

### 2.4 Analytical determinations

The oxidative balance was based on two different photometric tests: the concentration of peroxide, marker of lipid oxidation through the d‐ROMs FAST test (H&D srl) and the ferric reducing capacity as total antioxidant capacity through the *plasma antioxidant test* (PAT) test (H&D srl).

For the photometric reading, the dedicated FRAS5 system was used (H&D srl). The tests were performed in accordance with the manufacturer's instructions.

Both tests used sample plasma obtained by capillary blood collection. The capillary blood was taken using disposable sterile lancets and heparinized microvettes. The plasma was obtained from centrifugation (1600 *g* for 90 seconds) of the entire capillary blood sample.

### 2.5 d‐ROMs FAST test

The d‐ROMs FAST test is a colorimetric determination. The kit consists of R1 reagent, a condensed solution of N,N‐diethyl‐para‐phenylendiamine (chromogen), R2 reagent, acetic buffer pH 4,8, and R3 reagent, a ferric ion solution. Ten microliters of blood plasma is added to 1 mL of working solution and the mixture is poured onto R1 reagent. The working solution consists of 10 μL of R3 reagents in 1 mL of pre‐dosed R2 reagent. The photometric reading was taken at 505 nm after 150 seconds of incubation at 37°C.

This test is based on the Fenton reaction and the rate of coloration is proportional to the peroxide concentration. The results are expressed in U. Carr., where 1 U. Carr. = 0.08 mg/dL of H_2_O_2_. The normal range is between 250‐300 U. Carr.^20^

### 2.6 PAT test

*Plasma antioxidant test* is a colorimetric determination. The kit consists of R1 reagent, an alcohol solution of thiocyanate salt, and R2 reagent, an acid solution of ferric ion. Ten microliters of blood plasma is added to the working solution and the discoloration caused by the reduction of the ferric ion to the ferrous ion by antioxidants is proportional to the concentration of antioxidants. The working solution consists of 40 μL of R2 reagents in pre‐dosed R1 reagent. The photometric reading was taken at 505 nm after 60 seconds of incubation at 37°C. The results are expressed in U.Cor., where 1 U.Cor. = 1.4 μmol/L of vitamin C. The normal range is between 2200‐2800 U.Cor.^21^

### 2.7 Statistical analysis

The graphs and data analysis were produced using the software application Sigmaplot; the same graphs were produced by the software Origin 6.0. The appropriate *t* test (or Mann‐Whitney test if the normality was not demonstrated) and ANOVA (one‐way or two‐way) were used for the statistical analysis of differences between two or more groups. The Pearson correlation was used for the correlation analysis. The χ^2^ test was used for the analysis of the frequencies, where necessary. Any *P*‐values < .05 were considered significant. The identification of anomalous data was based on Cebysev's theorem of inequality. Any data in excess of the range: mean ± 3 times standard deviation were considered anomalous.

## 3 RESULTS

**FIGURE 1:**
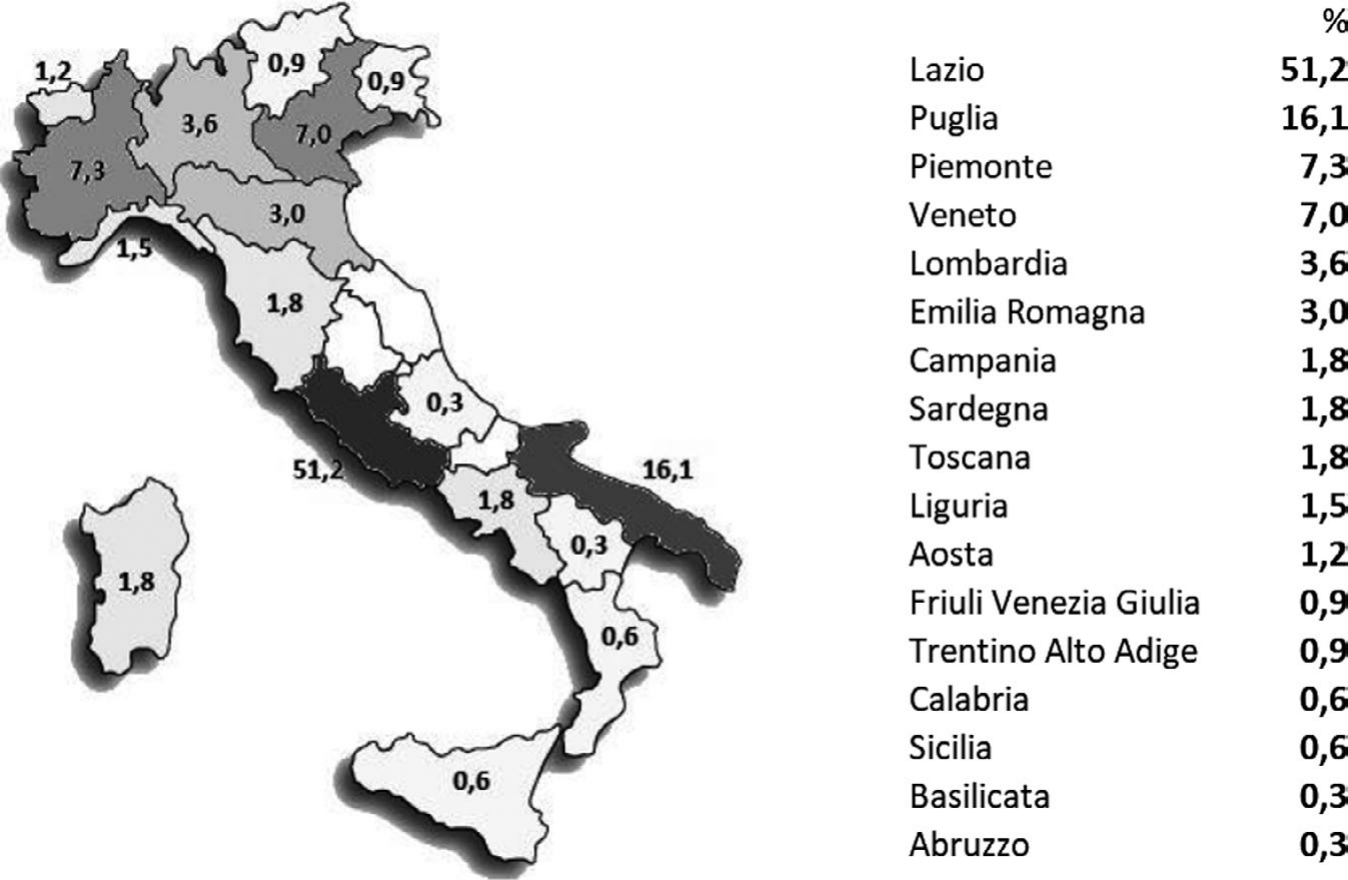
Percentage of volunteers for Italian region.

In this study 290 people were recruited from all over Italy (Figure 1). The sample analyzed consists of 105 males and 185 females. The mean age of the sample was 71 ± 7 years (min 60, max 92), in particular 74 ± 7 (min 60, max 92) for males and 70 ± 6 (min 60, max 88) for females. According to Cebysev's theorem of inequality, values which are too high or too low, probably caused by very specific and unknown clinical situations and not representative of the normal senior population, have been discarded.

In the male senior population, the mean d‐ROMs value is 412 ± 93 U. Carr. (min 186, max 661) and the mean PAT value is 2199 ± 295 U. Cor. (min 1282, max 2943).

**FIGURE 2:**
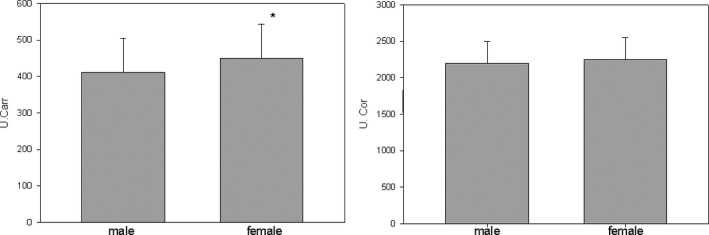
d‐ROMs (U. Carr.) and PAT (U. Cor.) values distribution in male and female senior population.

In the female senior population, the mean d‐ROMs value is 450 ± 93 U. Carr. (min 200, max 635) and the mean PAT value is 2246 ± 302 U. Cor. (min 1174, max 2990). The d‐ROMs values were significantly different between males and females (*P* = .001), but the PAT values (*P* = .211) were not significantly different (Figure 2).

From the data available it is not possible to establish a positive or negative correlation between the values of d‐ROMs and PAT and the age of the individual. The Pearson's *r* was calculated between d‐ROMs values and age, and between PAT values and age. In any analyzed case the correlation was lower than .1. This indicates that there is no significant correlation between age and oxidative stress marker values. The Pearson's *r* was calculated by dividing the sample by gender in order to exclude any possible remote correlation of age with d‐ROMs and PAT values. In order to examine deeply the trend of d‐ROMs and PAT values in relation to age, the samples were split into four age groups (60‐67; 68‐75; 76‐83; 84‐92) and the variance between groups was evaluated using ANOVA (two‐way; gender age group).

**FIGURE 3:**
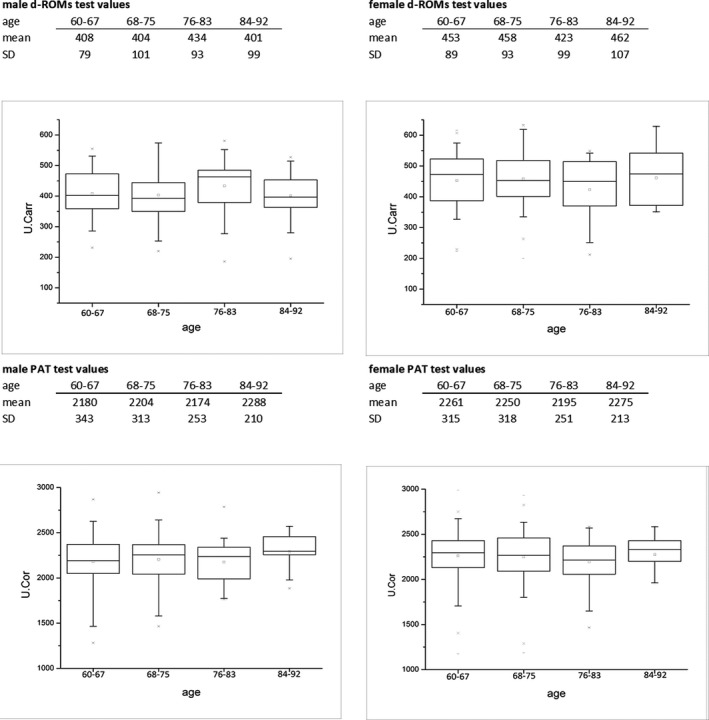
d‐ROMs and PAT test values divided by gender and age group.

The results of this statistical analysis are shown in Figure 3. For both oxidative stress marker values evaluated, no significant statistical difference between age groups was taken into consideration, *P* value is always >.05. The already known difference between genres of the level of d‐ROMs is the only significant difference (*P* = .01).

It has been verified that the average value of d‐ROMs and PAT deviate from the normal range, in particular the concentration of peroxides, but they are not statistically correlated with chronological age, as explained. To discover the distribution of the analyzed values, the population was divided according to gender and the values obtained divided into the reference categories. For the d‐ROMs test, the reference categories are as follows: lower value (<250 U. Carr.), normal value (250‐300 U. Carr.), higher value (301‐400 U. Carr.), very high value (>400 U. Carr.).^20^ For the PAT test, the reference categories are as follows: deficiency (<1800 U.Cor.), slight deficiency (1800‐2200 U.Cor.), normal value (2200‐2800 U.Cor.), excess (>2800 U.Cor.)^21^, ^22^, ^23^

**FIGURE 4:**
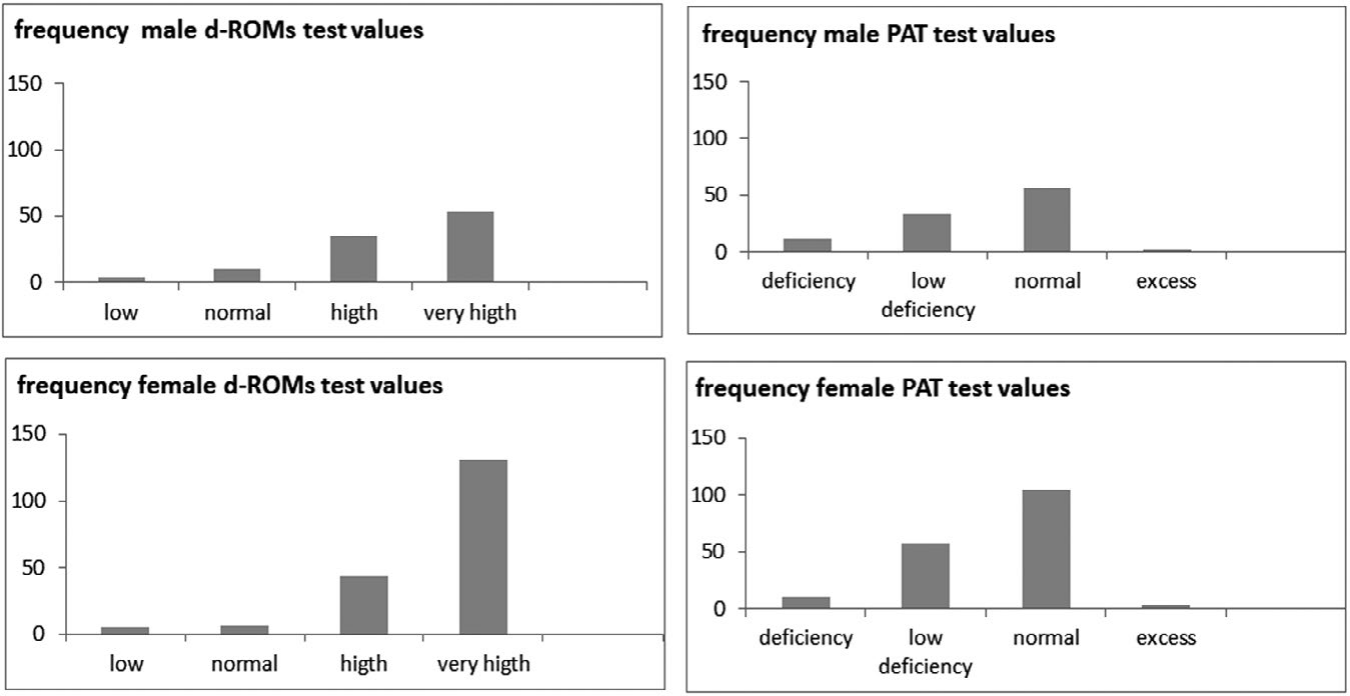
Frequency of d‐ROMs and PAT values in male and female elderly population.

Figure 4 shows the histograms of the population distribution, divided according to gender (105 men and 185 women), compared to the reference values categories. It is possible for d‐ROMs values to observe that the majority of the analyzed samples, both male and female, are included in the categories “high” (35 men and 43 women) or “very high” (54 men and 131 women). Meanwhile, for PAT values it was observed that the majority of the values are in the normal categories (56 men and 105 women), but there is a significant portion in the category “slight deficiency” (33 men and 58 women) (Figure 4).

**FIGURE 5:**
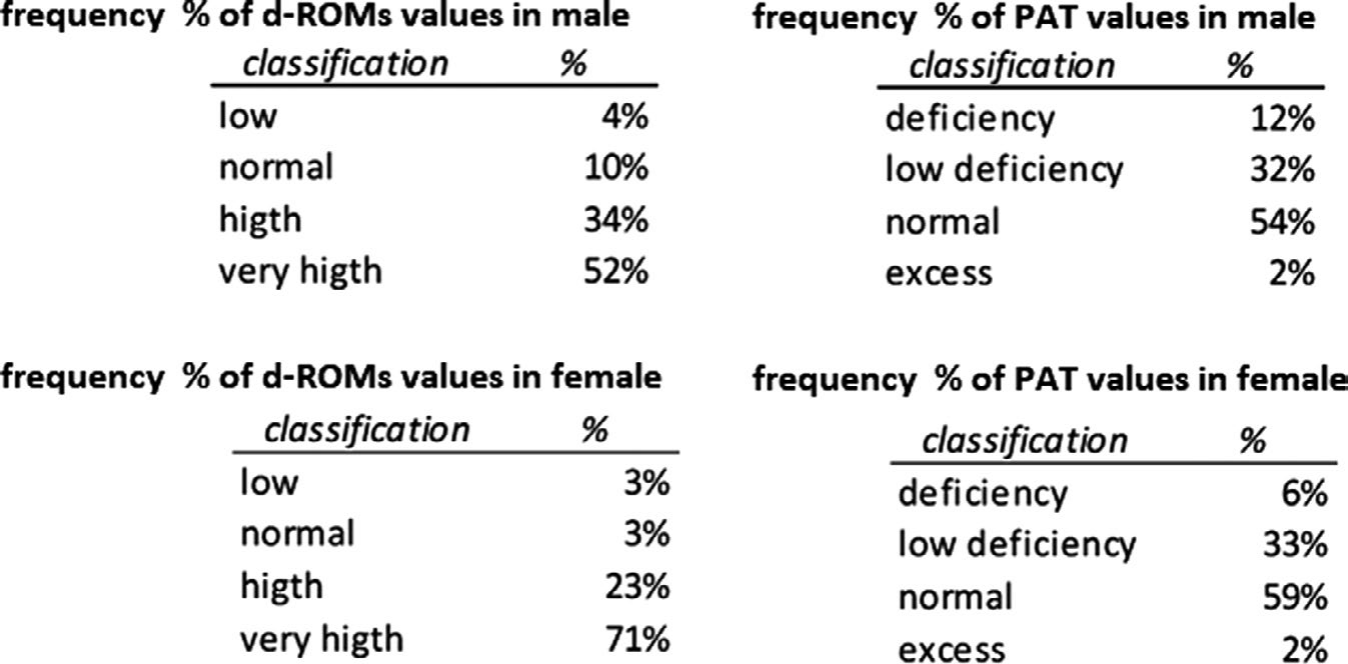
Frequency % of d‐ROMs and PAT values in male and female elderly population.

To summarize the results obtained: 34% of men and 23% of women have high values of d‐ROMs; 52% of men and 71% of women have a very high value of d‐ROMs; 32% men and 33% women have a slight deficiency of antioxidant reserve (Figure 5).

## 4 DISCUSSION

In this elderly population study, the two main markers of oxidative stress were evaluated. These markers allow the concentration of peroxides to be determined, as the oxidative damage index, and the iron‐reducing capacity, as the antioxidant reserve index. Antioxidant reserve represents the passive ability of the plasma to fight oxidative phenomena. It is well known that an accumulation of ROS can contribute to aging and to the pathogenesis of several age‐related conditions (i.e. cardiovascular diseases [CVDs], chronic obstructive pulmonary disease, chronic kidney disease, neurodegenerative diseases, and cancer), including sarcopenia and frailty.^4^, ^6^, ^24^

ROS are mainly generated in mitochondria. Harman was the first to suggest in the mid‐1950s that “free radicals produced during aerobic respiration have deleterious effects on cell components and connective tissues, causing cumulative damage over time that ultimately results in aging and death.”^2^

His theory was that mitochondria generate a significant amount of cellular energy and, through consumption of around 90% of the intracellular oxygen, set the limit on the lifespan. The premise of the mitochondrial free radical theory of aging is that mitochondria are both producers and targets of ROS. The latter attacks mitochondria and causes increased oxidative damage. As a consequence, damaged mitochondria progressively become less efficient, losing their ability to release oxygen molecules. Increased oxidative damage to the mitochondria culminates in an accumulation of age‐related dysfunctional mitochondria.^25^

This is why a prolonged OS condition and, in particular, increased levels of ROS and an insufficient antioxidant reserve could be very harmful for our organism.

A decline in normal antioxidant defense mechanisms has been observed in the aging brain and in the case of several neurodegenerative diseases.^19^ Such a decline increases the vulnerability of the brain to the deleterious effects of oxidative damage; the brains of patients with Alzheimer's disease showed reduced activity of antioxidant enzymes (SOD, catalase, glutathione peroxidase and glutathione reductase).^26^, ^27^

Different markers of oxidative stress in the aging brain have been broadly researched, for example 8‐OHdG (8‐hydroxy‐2′‐deoxyguanosine) as a biomarker of oxidative DNA damage in the aged brain.^28^, ^29^, ^30^

Changes in membrane fatty acid composition, as well as a decrease in the levels of polyunsaturated fatty acids (PUFAs, arachidonic acid) and an increase in monosaturated fatty acids, are related to aging. Arachidonic acids (AA) are abundant in the aging brain and are highly susceptible to ROS attack.

Oxidative damage to lipids can also occur indirectly through the production of highly reactive aldehydes. For example, the peroxidation of AA form malondialdehyde (MDA) causes DNA damage^31^, ^32^, ^33^, ^34^ or the peroxidation of linoleic acid which forms HNE (4‐hydroxy‐2‐nonenal), leading to a modification of protein activity.

HNE is able to migrate to sites that are distant from its formation and form covalent adducts with histidine, lysine, and cysteine residues in proteins. It has been shown that increased levels of HNE are found in Alzheimer's and Parkinson's disease.^35^, ^36^ Increasing evidence suggests that protein oxidation may be responsible for the gradual decline in physiological functioning that accompanies aging.

Increasing evidence associates aging and age‐related diseases with inflammation.^37^, ^38^, ^39^ Inflammation in the brain derives from the accumulation of reactive microglia in the degenerative areas.^40^, ^41^ The increased reactive microglia in the brain may be considered as an early event that leads to oxidative damage. Activated microglia are indeed the most abundant source of free radicals in the brain and release ROS.^42^ All these events, if prolonged over time, lead to oxidative damage and neuronal cell death in neurological diseases.^43^

CVD is a known pathology related to age and the oxidative stress condition. Its etiology derives from various risk factors such as hypercholesterolemia, hypertension, smoking, diabetes, poor diet, physical inactivity and others. Recent studies include ROS as a potential risk factor for CVD. It has been demonstrated that ROS lead to the oxidation of low‐density lipoprotein (OxLDL), which plays a key role in atherosclerosis pathogenesis.^44^, ^45^ ROS have also been implicated in congestive heart failure^46^ and are also the key intermediary related to vascular injury and organ dysfunction.^24^, ^47^ Recent studies demonstrated a correlation between oxidative stress increase and morbidity (all causes, CVD, cancer).^48^, ^49^, ^50^

In the present study it has been confirmed that in elderly people there is an OS condition characterized by increased levels of peroxides and a slight reduction in the antioxidant reserve.

Men and women revealed the same reduction in the antioxidant reserve, but women surprisingly showed a significantly higher d‐ROM value than men. This fact is consistent with the data obtained, where 70% of the women showed d‐ROM values higher than 400 U. Carr. against 50% of men.

The ages between men and women are considered differently, but in consideration of the fact that we have not found a correlation between age and markers, we do not believe that the origin of the difference between men and women is necessarily age.

There is growing evidence that the high concentration of ROS is connected with the majority of diseases (inflammatory, endocrinological, cardiological, neurological). The main problem in the study of ROS/disease correlation stems from the fact that it is not always clear whether the ROS are the cause, contributing factor or consequence of the disease.

This changing role of ROS in disease has considerable clinical relevance because if ROS are a cause or contributing factor, by acting on them it is possible to control, at least in part, the pathogenesis; if ROS are a consequence, their modulation only limits the propagation of free radical damage, but does not affect the pathogenesis.

The difference in the role of ROS as cause or consequence of disease is not always well defined. It is possible that the fact that they are both cause and effect allows the disease to develop a deleterious self‐sustaining cycle. One certain thing, supported by scientific literature, is that in different age‐related diseases there is an oxidative stress condition (Table 1).

**TABLE 1:** Oxidative stress condition in different age‐related disease

Neurological system	
Cognitive decline	62, 63
Alzheimer	35, 65, 66
Parkinson	68, 69
Cardiovascular system	
CAD (coronary artery diseases)	70, 71
Atherosclerosis	73, 74
Lung	
COPD (chronic obstructive pulmonary diseases)	75, 76
Ocular system	
Cataract	78, 79
Macular degeneration	54
Muscle‐skeletal system	
Osteoporosis	80, 81
Skin	
Skin disease	82, 83

In this study it has been shown that the level of ROS in elderly women is greater than that of elderly men. It is known that menopausal and post‐menopausal women have an imbalance of oxidative status, probably due to a decrease in estrogen levels. This reduction negatively affects the antioxidant defenses, causing an imbalance of the oxidative balance which favors ROS.^51^

This phenomenon could be one of the causes of the increased incidence in the female population over 60 of certain diseases in which the role of ROS has been confirmed. These include osteoporosis, in which a key role is played by H_2_O_2_ that causes dysfunction in osteoblast activity and reduces the activity of alkaline phosphatase;^52^, ^53^, ^54^, ^55^ a reduction in which increased ROS production is highlighted, due to chronic low‐intensity inflammation,^56^ and cardiovascular disease, where the oxidation of LDL plays a key role; in fact after menopause the risk of coronary heart disease doubles for women as the atherogenic lipids become greater than those of men.^57^

Despite that fact that in the present study, as expected, the majority of the senior population is in a condition of OS, surprisingly the levels of peroxides and antioxidants are not linearly correlated with age.

Based on Harman's theory,^2^ aging is mainly due to the decay of the redox compensation systems, but it has been hypothesized that this decay is not equal for all seniors.

Such decay is not dependent on simple increase in age, but on a more complex series of factors that might cause a deviation, sometimes a notable one.

It is undeniable that the values revealed that the senior population is more susceptible to an oxidative stress condition. This fact leads to the assumption that a person does not increase their oxidative stress condition by getting older, but only that in becoming old there is a greater chance of increasing oxidative stress condition.

**FIGURE 6:**
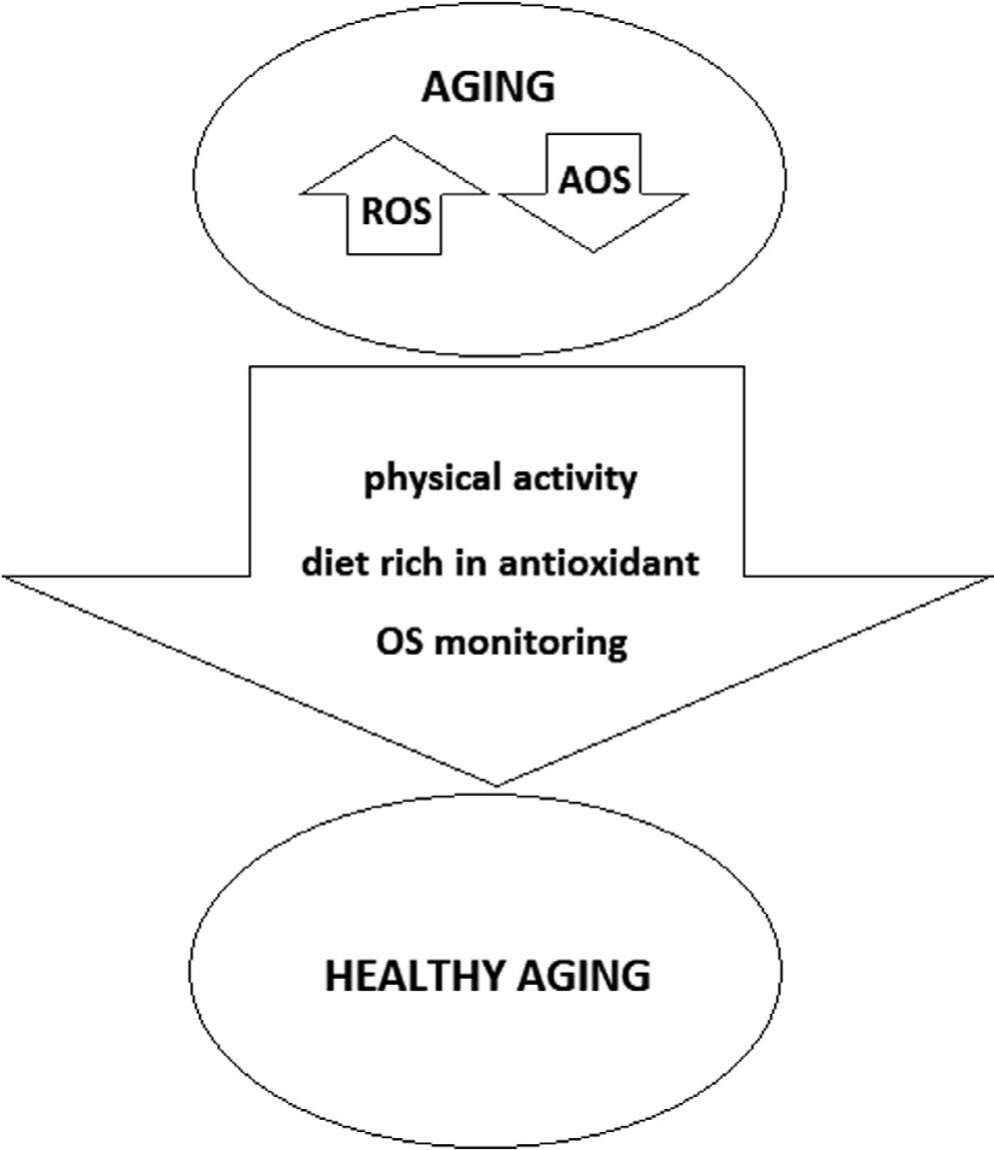
Summary of factors involved in healthy aging.

Based on the data which demonstrate that elderly people, especially women, are more prone to be in a condition of oxidative stress, it becomes essential to adopt an appropriate healthy lifestyle,^58^, ^59^ if necessary enhanced with specific food supplements or physiological modulators, and to monitor oxidative stress markers (Figure 6). Inactivity and aging are known to increase OS in skeletal muscle, leading to sarcopenia.^24^ In contrast, regular physical activity is an important determinant in maintaining an optimal state of health, reducing oxidative stress and preventing chronic diseases and CVDs.^24^, ^60^, ^61^

Epidemiological evidence suggests that a diet rich in fruit and vegetables and an appropriate lifestyle (reduction of smoking, reduction of alcohol consumption and adequate physical activity) may help to prevent certain age‐related diseases (cardiovascular disease, stroke, diabetes, Parkinson's disease, and Alzheimer's disease)^24^ (Figure 6). Equally important is the modulation of antioxidant intake through food supplements, since an excessive concentration of these can paradoxically act as a pro‐oxidant and promote the redox imbalance. An antioxidant, in fact, once it has given up its electron or hydrogen, becomes a pro‐oxidant (redox system), so if it is administered in excessive amounts it can have the opposite effect.

Our conclusion is that the deterioration of oxidative status is not linear, indicating that the main cause of this decline is not the passing of time (years) but the multiplication of the possibilities of adverse events (diseases, dietary habits, alteration of cellular functions).

All these adverse events may, to a greater or lesser degree, influence the progression of aging and the oxidative conditions. The assessment of OS conditions should be first step for at least improving the quality of aging progression.

Monitoring can highlight any increase or decrease in ROS and/or antioxidant reserves and the specialist will be able to advise on proper nutrition, adequate exercise or adequate decisions by modulating this suggestion according to individual needs, in order to guarantee a healthy aging. A limitation of this study was the self‐compilation of the questionnaire and the impossibility of verifying the real clinical status of the subjects. This is the reason why some data related to diseases and its relation with OS, have not been reported. Further studies will be necessary in order to evaluate the correlation between OS and the different age‐related diseases.

## CONFLICTS OF INTEREST

The tests were offered by H&D srl for an awareness‐raising project on oxidative stress and aging. Nothing to disclose.

## AUTHOR CONTRIBUTIONS

D. Gorni and A. Finco contributed to the conception and design; contributed to acquisition, analysis and interpretation; drafted the manuscript; critically reviewed the manuscript; gave final approval; and agree to be accountable for all aspects of the work, ensuring integrity and accuracy.

## ETHICAL APPROVAL

All procedures performed in this study involving human participants are in accordance with Helsinki declaration and its later amendments or comparable ethical standards.
